# Nomogram establishment for gastrointestinal bleeding prediction in ICU patients with traumatic brain injury based on the MIMIC-IV database

**DOI:** 10.3389/fmed.2025.1523535

**Published:** 2025-04-14

**Authors:** Ziming Huang, Hengfa Ge, Ying Sun

**Affiliations:** ^1^The Affiliated Huaian No.1 People’s Hospital of Nanjing Medical University, Huai'an, China; ^2^Jiangsu Food and Pharmaceutical Science College, Huai'an, China

**Keywords:** gastrointestinal bleeding, traumatic brain injury, MIMIC-IV, shock index, ROC, DCA, nomograms

## Abstract

**Objectives:**

This study aimed to develop a robust nomogram for predicting the occurrence of gastrointestinal bleeding (GIB) in patients with traumatic brain injury (TBI) during their ICU stay, thereby facilitating the optimization of intervention strategies and enabling personalized treatment approaches.

**Methods:**

Patient data were extracted from the publicly available MIMIC-IV (Medical Information Mart for Intensive Care IV) database. In this retrospective cohort study, a total of 2,774 patients with traumatic brain injury (TBI) were included. A 7:3 ratio was applied to allocate patients into the training and validation cohorts. A LASSO logistic regression model was constructed using the training set to identify potential predictors of gastrointestinal bleeding (GIB). The selected features were subsequently utilized to develop a nomogram model. The performance of the nomogram was evaluated using receiver operating characteristic (ROC) curves, calibration curves, and decision curve analysis (DCA).

**Results:**

A nomogram model comprising six variables—gender, blood urea nitrogen (BUN), Shock Index (SI), albumin, SOFA score, and diabetes mellitus—was developed. These variables were identified as independent risk factors for gastrointestinal bleeding (GIB) in patients with traumatic brain injury (TBI) (*p* < 0.05). The area under the receiver operating characteristic curve (AUC) for the derivation cohort and validation cohort was 0.8541 (95% CI: 0.833 to 0.911) and 0.8381 (95% CI: 0.752 to 0.863), respectively. The calibration curve demonstrated good agreement between the predicted probabilities and actual observations, while decision curve analysis (DCA) highlighted the clinical utility of the predictive model.

**Conclusion:**

This study developed a predictive model for GIB in patients with TBI, which may assist clinicians in early identification of high-risk patients and help mitigate the burden of GIB in susceptible populations.

## Introduction

Traumatic brain injury (TBI) is a leading cause of disability and mortality worldwide. Estimates suggest that between 64 and 74 million individuals globally experience TBI annually (1). The consequences of TBI vary widely, ranging from mild concussions to severe outcomes such as coma or death, depending on the magnitude of force impacting the skull and intracranial structures (2, 3). The burden of TBI is substantial, affecting both individuals and society due to its high prevalence, long-term consequences, loss of workforce productivity, strain on healthcare systems, and its impact on family dynamics, social participation, and health inequalities (4, 5).

Patients with TBI frequently develop gastrointestinal bleeding (GIB), which can significantly increase mortality rates (6, 7). GIB arises from mucosal barrier disruption and excessive gastric acid secretion, with proposed mechanisms including hypoperfusion, microcirculatory disturbances, ischemia, pathological luminal acidosis, proinflammatory states, hypovolemia, and shock (8). To date, while several studies have explored treatment strategies for TBI and its complications, no systematic investigation has specifically focused on predicting the risk of GIB among TBI patients in the intensive care unit (ICU).

Nomograms are increasingly recognized as robust tools for clinical prediction, offering advantages over traditional scoring systems (9, 10). By integrating multiple clinical and demographic factors, nomograms provide personalized risk estimates in an accessible graphical format. They accommodate both continuous and categorical variables, enabling more precise predictions tailored to individual patient needs (11). These models have demonstrated significant potential across various clinical settings, serving as effective risk stratification tools and integral components of modern evidence-based medical decision-making (12).

Therefore, this study aims to develop a nomogram based on the Medical Information Mart for Intensive Care IV (MIMIC-IV) database to integrate multiple independent risk factors for GIB in TBI patients within the ICU setting. This tool will facilitate individualized patient management and support the development of evidence-based prevention and intervention strategies.

## Materials and methods

### Medical Information Mart for Intensive Care IV

The MIMIC-IV database is a publicly accessible, multiparametric critical care database provided by MIT. It includes comprehensive data from patients who were treated in the Intensive Care Unit (ICU) at Beth Israel Deaconess Medical Center (BIDMC) between 2008 and 2019 (13). This project was approved by the Institutional Review Boards of both MIT and BIDMC and was granted an exemption from the requirement for informed consent. Additional information can be accessed at https://physionet.org/content/mimiciv/2.2/ (14). The MIMIC-IV dataset utilized in this retrospective analysis comprises accurate medical records that are freely accessible to researchers. All personally identifiable information within the database has been systematically deidentified and replaced with randomized codes to ensure patient anonymity. As such, the use of publicly available databases does not require patient-informed consent or additional ethical approval.

### Patient selection

**Figure 1:**
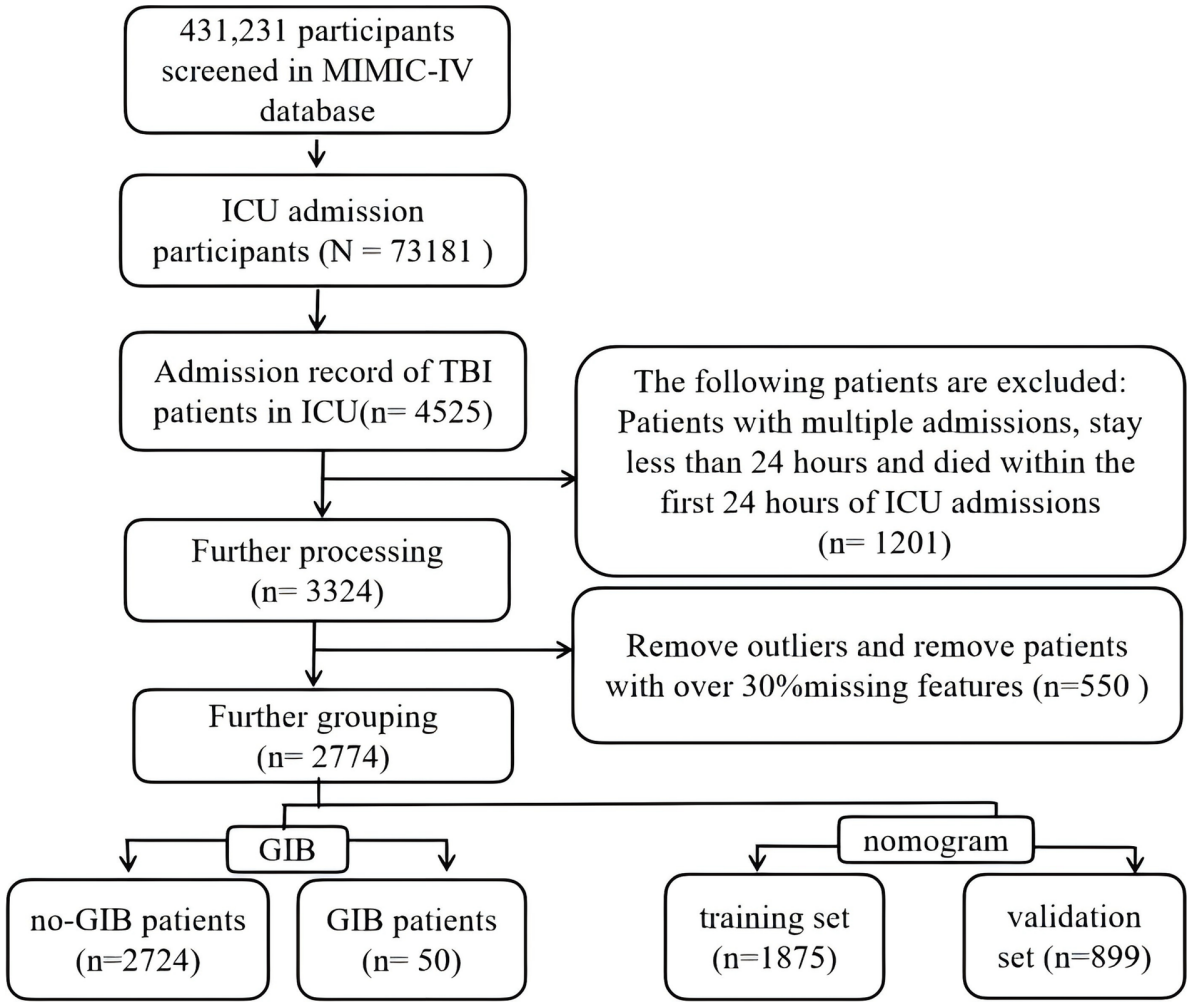
Flow chart of selection. ICU, intensive care unit; MIMIC-IV, Medical Information Mart for Intensive Care IV; GIB, gastrointestinal bleeding; TBI, traumatic brain injury.

TBI cases were identified by querying the ICD codes in PgAdmin software using the following codes: 800–804, 851, and S06. Patients with GIB were identified through a search of the following ICD codes: 531, 534, 535, K24, K25, and K28. Patients who were not admitted to the ICU for the first time, had an ICU stay shorter than 1 day, or died within 1 day of ICU admission were excluded from the analysis. Excluding patients with ICU stays ≤ 24 h in a study on GIB in TBI patients is methodologically justified, with key rationales including: Mitigation of Immortal Time Bias, Time-Dependent Medical Interventions, Pathophysiological Timeline, Control of Confounding Factors and Data Standardization Lab parameters. Ultimately, a total of 2,774 patients were included and divided into two groups: the GIB group and the Non-GIB group. For the purpose of constructing the nomogram, all subjects were randomly allocated to either a training set or a testing set at a ratio of 7:3 (Figure 1).

### Data collection

The MIMIC-IV database consists a total of 91 tables include 61 tables in MIMIC-IV_derived menu, 22 tables in MIMIC-IV_hosp menu and 9 tables in MIMIC-IV_icu menu, each meticulously documenting specific details related to demographic characteristics, vital signs, severity scores, laboratory indicators, complications and treatment information (15).

Demographic data included age, gender, race, weight. Vital signs included systolic blood pressure (SBP), diastolic blood pressure (DBP), mean arterial pressure (MBP), respiratory rate (RR), temperature, Oxygen saturation (SPO2) and blood glucose. Severity scores included Sequential Organ Failure Assessment Score (SOFA), Simplified Acute Physiology Score II (SAPS II) and Glasgow Coma Scale (GCS). Laboratory indicators included Serum sodium, potassium, chloride, creatinine, albumin, hemoglobin, calcium, blood urea nitrogen (BUN), white blood cells (WBC), platelets (PLT), PaO2/FiO2 ratio, BE, lactate. Treatment information include mechanical ventilation and the use of vasopressors (norepinephrine, epinephrine, phenylephrine, dopamine, and vasopressin) within 24 h of ICU admission, Vital signs and laboratory indicators were collected within 24 h of ICU admission. Comorbidities were searched through PgAdmin software including myocardio_infarction, sepsis, Hypertension (HPB), Chronic Obstructive Pulmonary Disease (COPD), acute kidney injury (AKI), Multiple rib fractures (MRF), asthma, pelvic fracture, diabetes. For indicators with multiple measurements, the most severe measurement was used for data analysis.

### Ethics approval and consent to participate

Ethical approval was obtained from the Institutional Review Boards (IRBs) of both the Massachusetts Institute of Technology (Cambridge, MA, United States) and Beth Israel Deaconess Medical Center (Boston, MA, United States). The author of this study completed the Collaborative Institutional Training Initiative (CITI) program and successfully passed the examinations for “Data or Specimens Only Research” and “Conflicts of Interest” (CITI ID: 12765719).

### Statistical analysis

Continuous data were presented as the median and interquartile range (IQR) and analyzed using the Wilcoxon–Mann–Whitney test. Categorical data were expressed as percentages (%) and compared using the chi-square test. A univariate logistic regression was employed to analyze the training set to identify potential variables associated with adverse outcomes. To address collinearity issues and determine the most relevant risk factors predicting GIB in patients with TBI, an initial screening was conducted via LASSO regression analysis. Variable Selection Workflow Clarification: The predictors in the final nomogram were rigorously selected through a three-step process: Step 1: univariate screening j, variables with *p* < 0.1 in univariate analysis were retained to avoid prematurely excluding potential confounders; Step 2: LASSO Regression for Dimension Reduction, To address multicollinearity and overfitting, we applied LASSO regression with 10-fold cross validation. LASSO penalizes the absolute size of regression coefficients, automatically shrinking non-informative variables to zero while retaining clinically/biologically meaningful predictors. Variables with non-zero coefficients after penalization were selected for further analysis; Step 3: Multivariable Validatio, The LASSO-selected variables were validated using logistic regression to estimate adjusted odds ratios.

The variables identified through LASSO regression were utilized to construct a nomogram. The discriminative performance of the nomogram model was evaluated using the area under the curve (AUC) derived from receiver operating characteristic (ROC) analysis. A calibration curve was generated via 1,000 bootstrap resamplings, and the calibration accuracy was assessed using a Cox regression model. Decision curve analysis (DCA) was performed to evaluate the clinical utility of the nomogram in both the training and validation cohorts.

Statistical analyses were performed using Stata software (version 16, StataCorp, College Station, TX, United States), while graphical representations were generated with R (version 3.6.1, R Foundation for Statistical Computing, Vienna, Austria). The R packages employed included haven, dplyr, rms, ROCR, regplot, rmda, and glmnet.

### Management of missing data

The “scatter” command in Stata software was utilized to detect and handle outlier values, which were subsequently excluded and coded as missing values. Variables with more than 15% missing values were omitted from the analysis. This 15% missingness threshold was established based on precedent in peer studies, specifically as implemented in the high-impact methodology by Liu et al. (16), who analyzed the same MIMIC-IV database: “Variables with missing values exceeding 15% were excluded to ensure reliable multivariable modeling.” For variables with missing values between 5% and 15%, imputed values were generated using an appropriate random sample drawn from their predicted distribution. Conversely, for variables with missing values below 5%, the missing values were substituted with the mean of the respective variable.

### Reporting standards

This study adheres to the TRIPOD-Cluster checklist (17) for transparent reporting of prediction models developed with clustered data.

## Results

### Patients selection and baseline characteristics

A total of 4,525 first ICU admission records for TBI were retrieved from the MIMIC-IV database. Following the exclusion criteria, which included patients with multiple admissions, those with a hospital stay duration of less than 24 h, and individuals who died within the first 24 h of ICU admission (*n* = 1,201), the remaining records were analyzed. Ultimately, we obtained 3,324 admission records for patients with TBI. Among these cases, an additional 550 patients were excluded due to incomplete clinical data or treatment abandonment. Consequently, a total of 2,774 individuals were included in our cohort, of whom 50 experienced gastrointestinal bleeding (GIB). Table 1 presents the baseline characteristics of all participants, categorized into the Non-GIB group and the GIB group. Compared with the Non-GIB group, patients in the GIB group exhibited a higher shock index, indicating more severe trauma. Additionally, within the first 24 h of admission, the GIB group demonstrated significantly elevated Sequential Organ Failure Assessment (SOFA) scores compared to the Non-GIB group (*p* < 0.05), suggesting more severe organ dysfunction in this subgroup. Furthermore, individuals in the GIB group had lower albumin and hemoglobin levels but higher creatinine and blood urea nitrogen levels, and they were more likely to have diabetes status.

**Table 1:** Comparison of baseline data between Non-GIB and GIB cohort.

**Variables**	**Non-GIB**	**GIB**	**Total**	**C/Z**	***p* for value**
	**(*N*=2724)**	**(*N*=50)**	**(*N*=2774)**		
Characteristics					
Gender					
Male	1654 (60.7%)	38 (76.0%)	1692 (61.0%)	4.8187	0.028
Female	1070 (39.3%)	12 (24.0%)	1082 (39.0%)		
Age (years)	64.5 [49.4, 78.9]	62.8[53.9, 62.2]	64.4 [49.5, 78.8]	0.1926	0.8473
Race				22.4731	<0.001
Asian	74 (2.7%)	3 (6.0%)	77 (2.8%)		
Black	180 (6.6%)	10 (20.0%)	190 (6.8%)		
White	1709 (62.7%)	31 (62.0%)	1740 (62.7%)		
Hispanic	97 (3.6%)	3 (6.0%)	100 (3.6%)		
Other	664 (24.4%)	3 (6.0%)	667 (24.0%)		
Weight (kg)	75.2 [63.4, 88.5]	74.7[64,87.7]	75.0 [63.5, 85.5]	0.157	0.875
Severity score					
GCS	14.0 [13.0, 14.0]	14.0 [13.00, 15.0]	14.0 [13.0, 14.0]	-0.947	0.3437
SOFA	3 [2, 5]	4.00 [3, 7]	3 [2, 5]	-3.508	0.0005
SAPSII	31.0 [23, 39]	32.5 [24, 40]	31.0 [23, 39]	-0.552	0.5808
Vital signs					
SI	1.01 [0.86, 1.23]	1.42 [1.25, 1.63]	1.02 [0.86, 1.24]	-2.9942	0.0028
Heart rate( times/min)	99.0 [87,114]	105 [95, 118]	99.0 [87, 114]	-1.8469	0.0649
SBP (mm Hg)	97 [86, 108]	95 [84, 108]	97 [86 108]	0.5463	0.5849
DBP (mm Hg)	49 [42, 56]	50 [42, 60]	49 [42,56]	-1.8584	0.0632
MBP (mm Hg)	63 [55, 71]	61.5 [53, 67]	63 [55, 71]	0.5678	0.5702
Resp rate (times/min)	26 [23, 30]	27 [23, 32]	26 [23, 30]	-1.2351	0.2169
Temperature (°C)	36.5 [36.39, 36.78]	36.5 [36.22, 36.67]	36.6[36.39,36.78]	1.5113	0.1308
Spo2	94 [91, 96]	93.0 [90, 95]	94 [91, 96]	0.597	0.5505
Glu (mg/dL)	143 [117, 182]	143 [113, 175]	143 [117, 182]	0.1393	0.8892
Sodium (mmol/L)	139 [137, 142]	139 [136, 141]	139 [137.142]	0.2819	0.778
Potassium (K/μL)	4.00 [3.7, 4.3]	4.00 [3.7, 4.6]	4.00 [3.7, 4.3]	-0.7069	0.4797
Chloride (mmol/L)	104 [101,107]	106 [101, 109]	104 [101, 107]	-0.6729	0.5011
Creatinine (mg/dL)	0.8 [0.7, 1.1]	0.900 [0.7, 1.4]	0.8 [0.7, 1.1]	-5.6636	<0.001
Albumin (g/dL)	3.50 [3.5, 3.5]	3.30 [3.1, 3.3]	3.50 [3.5, 3.5]	3.7929	0.0002
Haemoglobin (g/dL)	11.7 [10.2, 13]	11.1 [8.6, 12.8]	11.7 [10.2, 13]	2.1782	0.0295
Calcium (mmol/L)	1.09 [1.09,1.09]	1.09 [1.09,1.09]	1.09 [1.09,1.09]	-0.3413	0.7329
BUN (mg/dL)	15.0 [11, 20]	17.5 [12, 35]	15.0 [11, 21]	-4.0857	<0.001
WBC (K/μL)	10.4 [7.9, 13.7]	10.3 [6.7,14.1]	10.4 [7.9, 13.7]	-0.1147	0.9087
Platelet (K/μL)	193 [152, 242]	173 [133, 230]	193 [152, 242]	0.9856	0.3244
P/F	327.5 [327.5 , 327.5 ]	317.5[317.5, 317.5]	327.5[327.5, 327.5 ]	1.1962	0.2317
BE (mmol/L)	-1.1 [-1.1.-1.1]	-1.2 [-1.2.-1.1]	-1.1 [-1.1.-1.1]	-0.3761	0.7069
Lactic acid (mmol/L)	1.9 [1.9, 1.9]	2.20 [2.2, 2.2]	1.9 [1.9, 1.9]	-1.2538	0.2100
Comorbidities					
Myocardio infarction					
NO	2678 (98.3%)	50 (100%)	2728 (98.3%)		
YES	46 (1.7%)	0 (0%)	46 (1.7%)	0.8586	0.354
Sepsis					
YES	1108 (40.7%)	25 (50.0%)	1133 (40.8%)		
NO	1616 (59.3%)	25 (50.0%)	1641 (59.2%)	1.7668	0.184
HBP					
YES	1144 (42.0%)	25 (50.0%)	1169 (42.1%)		
NO	1580 (58.0%)	25 (50.0%)	1605 (57.9%)	1.2897	0.256
COPD					
YES	30 (1.1%)	3 (6.0%)	33 (1.2%)		
NO	2694 (98.9%)	47 (94.0%)	2741 (98.8%)	0.0888	0.766
AKI					
NO	1088 (39.9%)	19 (38.0%)	1107 (39.9%)		
YES	1636 (60.1%)	31 (62.0%)	1667 (60.1%)	0.0772	0.781
Multiple rib fracture					
YES	94 (3.5%)	1 (2.0%)	95 (3.4%)		
NO	2630 (96.5%)	49 (98.0%)	2679 (96.6%)	0.3125	0.576
Asthma					
NO	2561 (94.0%)	47 (94.0%)	2608 (94.0%)		
YES	163 (6.0%)	3 (6.0%)	166 (6.0%)	0.001	0.996
Pelvic fracture					
YES	706 (25.9%)	9 (18.0%)	715 (25.8%)		
NO	2018 (74.1%)	41 (82.0%)	2059 (74.2%)	1.6089	0.205
Diabetes					
YES	586 (21.5%)	17 (34.0%)	603 (21.7%)		
NO	2138 (78.5%)	33 (66.0%)	2171 (78.3%)	4.5005	0.034
Mechanical ventilation					
YES	1129 (41.4%)	18 (36.0%)	1147 (41.3%)		
NO	1595 (58.6%)	32 (64.0%)	1627 (58.7%)	0.6006	0.438

Medians and interquartile ranges (25th and 75th percentiles) were computed for continuous variables, and frequencies and percentages for categorical variables. The Wilcoxon rank-sum test was used to compare group differences for continuous variables, and Chi-square tests for categorical variables. GIB, gastrointestinal bleeding; APSIII, acute physiology score III; GCS, Glasgow Coma Score; SOFA, sequential organ failure assessment; SI, shock index; MBP, mean blood pressure; SBP, systolic blood pressure; DBP, diastolic blood pressure; SpO2, pulse oximetry-derived oxygen saturation; PaO2: arterial partial pressure of oxygen; WBC, white blood cell count; BUN: blood urea nitrogen; WBC, white blood cells; PLT, platelets; HBP, high blood pressure;COPD, chronic obstructive pulmoriary disease; AKI, acute kidney injury.

### Univariate and multivariate logistic regression analysis of GIB in TBI

We performed a univariate analysis of the overall clinical baseline characteristics using the training set. The risk factors identified through the univariate analysis were subsequently included in the multivariate regression analysis. The results of the univariate and multivariate logistic regression analyses for GIB in patients with TBI are presented in Table 2. Multivariate logistic regression analysis revealed that gender (OR: 2.612, 95% CI 1.132 to 6.123, *p* = 0.021), SOFA score (OR: 1.133, 95% CI 1.051 to 1.179, *p* = 0.033), shock index (SI) (OR: 1.122, 95% CI 1.097 to 1.370, *p* = 0.031), blood urea nitrogen (BUN) (OR: 1.015, 95% CI 1.010 to 1.0203, *p* = 0.048), albumin level (OR: 0.444, 95% CI 0.222 to 0.887, *p* = 0.022), and diabetes (OR: 1.388, 95% CI 1.134 to 1.189, *p* = 0.01) were independent risk factors for GIB in patients with TBI.

**Table 2:** Univariate and multivariate logistic regression analyses in the training set.

**Variables**	Odds Ratio	Std. Err.	z	*P*>z	[95% Conf.Interval]	
Gender	2.789549	1.182193	2.42	0.015	1.215626	6.401296
Age (years)	0.9964305	0.008294	-0.43	0.667	0.9803064	1.01282
Race	1.17668	0.1686213	1.14	0.256	0.8885442	1.558252
Weight (kg)	0.9976723	0.0080881	-0.29	0.774	0.9819453	1.013651
Severity score						
GCS	1.008332	0.0676074	0.12	0.902	0.8841619	1.149941
SOFA	1.102494	0.0490773	2.19	0.028	1.010381	1.203005
SAPSII	1.000274	0.0132948	0.02	0.984	0.9745528	1.026674
Vital signs						
SI	1.139447	0.0862905	1.72	0.085	0.9822737	1.321769
Heart rate (times/min)	1.013706	0.0077448	1.78	0.075	0.99864	1.029
SBP (mm Hg)	1.005435	0.0101348	0.54	0.591	0.9857665	1.025497
DBP (mm Hg)	1.041256	0.014799	2.84	0.004	1.012651	1.070669
MBP (mm Hg)	1.005392	0.0121738	0.44	0.657	0.981813	1.029538
Resp rate (times/min)	1.040538	0.0250208	1.65	0.098	0.9926362	1.090752
Temperature (°C)	1.072082	0.2734766	0.27	0.785	0.6502729	1.767504
Spo2	0.9928995	0.020994	-0.34	0.736	0.9525929	1.034912
Glu (mg/dL)	0.9982288	0.0026378	-0.67	0.502	0.9930722	1.003412
Sodium (mmol/L)	0.9818912	0.0336905	-0.53	0.594	0.9180304	1.050194
Potassium (K/μL)	1.123759	0.2565167	0.51	0.609	0.71841	1.757819
Chloride (mmol/L)	1.018351	0.0307986	0.6	0.548	0.9597414	1.08054
Creatinine (mg/dL)	1.252721	0.0868496	3.25	0.001	1.093558	1.43505
Albumin (g/dL)	0.3832315	0.1182532	-3.11	0.002	0.2093182	0.7016416
Haemoglobin (g/dL)	0.8832664	0.0695811	-1.58	0.115	0.7568967	1.030735
Calcium (mmol/L)	3.363832	6.444554	0.63	0.527	0.0787174	143.7467
BUN (mg/dL)	1.01437	0.0063027	2.3	0.022	1.002091	1.026798
WBC (K/μL)	0.9948898	0.0282201	-0.18	0.857	0.9410889	1.051767
Platelet (K/μL)	0.9981236	0.0022152	-0.85	0.397	0.9937913	1.002475
P/F	0.9998711	0.001673	-0.08	0.939	0.9965973	1.003156
BE (mmol/L)	1.100492	0.0774617	1.36	0.174	0.9586773	1.263286
Lactic acid (mmol/L)	1.106707	0.1402694	0.8	0.424	0.863271	1.41879
Myocardio infarction	Ref.					
Sepsis	1.50545	0.5069108	1.21	0.224	0.7781298	2.912597
HBP	1.712307	0.5799879	1.59	0.112	0.8815866	3.325816
COPD	0.5303952	0.5408426	-0.62	0.534	0.0718839	3.91352
AKI	0.9341793	0.3189248	-0.2	0.842	0.4784469	1.824008
Multiple rib fracture	0.7797802	0.796949	-0.24	0.808	0.1052033	5.77983
Asthma	0.9428114	0.6923396	-0.08	0.936	0.2235412	3.976419
Pelvic fracture	0.461846	0.2239323	-1.59	0.111	0.1785586	1.194576
Diabetes	1.587972	0.5815312	1.26	0.207	0.7746839	3.255078
Mechanical ventilation	0.7227394	0.2578162	-0.91	0.363	0.3592034	1.454196
Multivariate logistic regression analyses						
Gender	2.612345	1.1345199	2.21	0.021	1.132283	6.123193
SOFA	1.133345	0.0380269	1.13	0.033	1.0516857	1.179584
SI	1.122326	0.0778446	1.61	0.031	1.0970852	1.370222
BUN (mg/dL)	1.005304	0.0076341	0.7	0.0486	1.010453	1.020379
Diabetes	1.388721	0.1369208	4.23	0.001	1.134435	1.189244
Albumin (g/dL)	0.423878	0.1567515	-2.3	0.032	0.332587	0.897322

GIB, gastrointestinal bleeding; APSIII, acute physiology score III; GCS, Glasgow Coma Score; SOFA, sequential organ failure assessment; SI, shock index; MBP, mean blood pressure; SBP, systolic blood pressure; DBP, diastolic blood pressure; SpO2, pulse oximetry-derived oxygen saturation; PaO2: arterial partial pressure of oxygen; WBC, white blood cell count; BUN: blood urea nitrogen; WBC, white blood cells; PLT, platelets; HBP, high blood pressure;COPD, chronic obstructive pulmoriary disease; AKI, acute kidney injury.

### LASSO logistic regression variable screening and nomogram establishment

**Figure 2:**
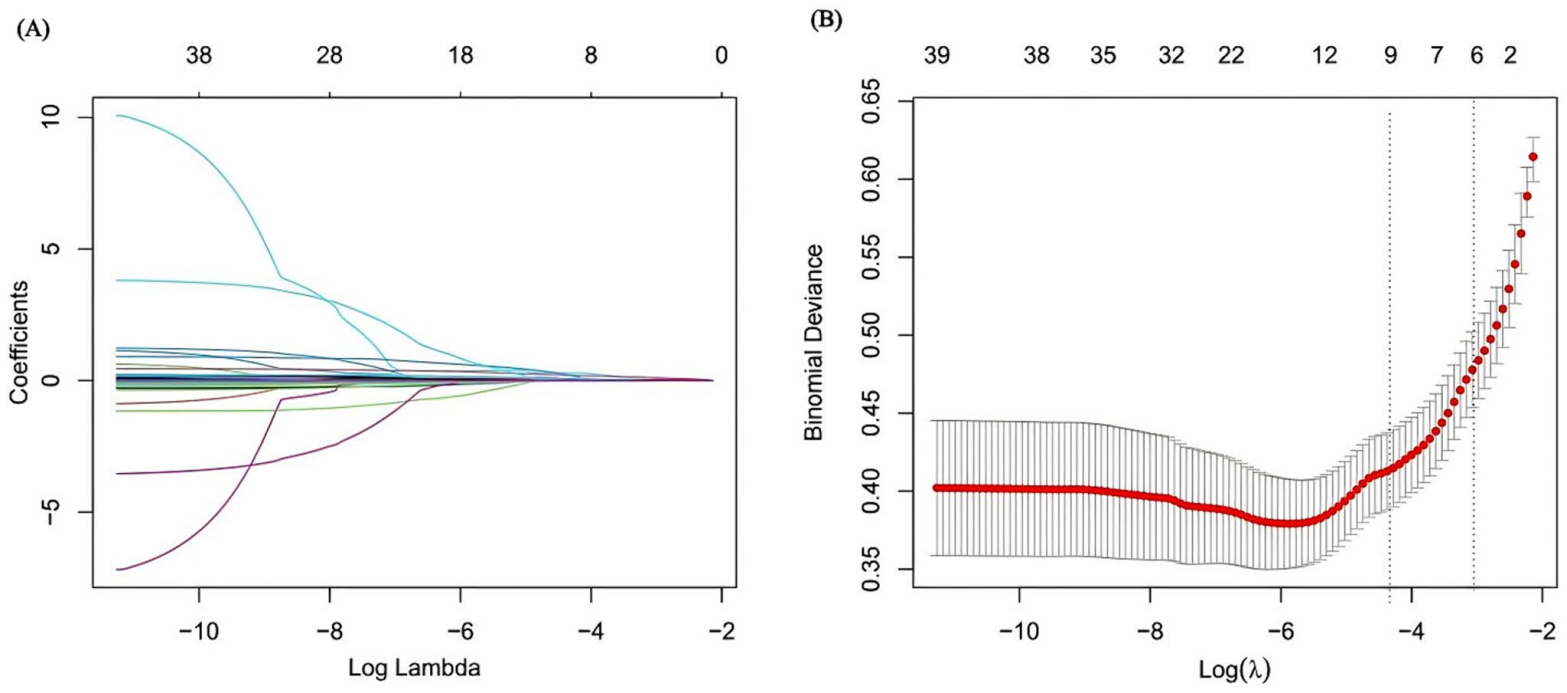
Lasso regression variable trajectories, the x-coordinate at the top of figure indicates the number of variables (dummy variables). **(A)** Each curve with different colors represents the change trajectory of each independent variable coefficient, the y-axis is the coefficient value; the upper x-axis is the number of non-zero coefficients in the LASSO model; **(B)** Represented the cross-validation result with different λ value, the left dot line represented lambda.min which was the lowest λ of minimum mean cross-validated error, the right dot line represented the lambda.1se.

**Figure 3:**
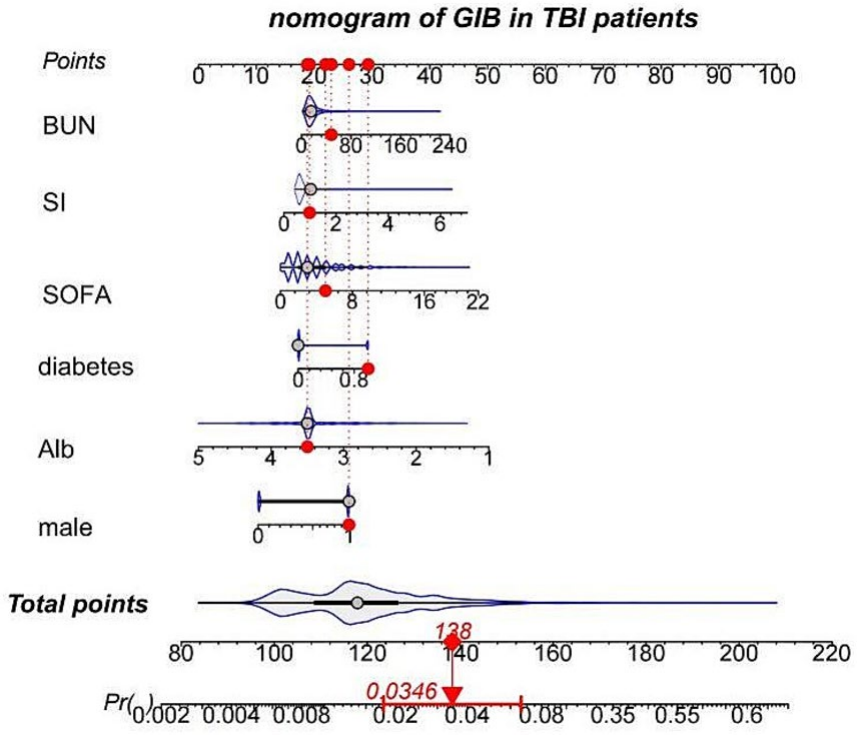
The predictive nomogram for the incidence of GIB with TBI. GIB, gastrointestinal bleeding; TBI, traumatic brain injury; SOFA, sequential organ failure assessment; SI, shock index; Alb, Albumin.

To ensure the accuracy of the model, further feature selection was conducted using LASSO regression. Figure 2 displays the results of variable screening performed by LASSO regression. The six identified risk factors included gender, SOFA score, shock index (SI), blood urea nitrogen (BUN), albumin level, and diabetes status. Based on these selected features, a nomogram was constructed, as illustrated in Figure 3. The total points can be calculated by summing the scores assigned to each variable within the nomogram. Consequently, the probability corresponding to this total score represents the likelihood of gastrointestinal bleeding (GIB) in patients with traumatic brain injury (TBI).

### Validation of the nomogram model

**Figure 4:**
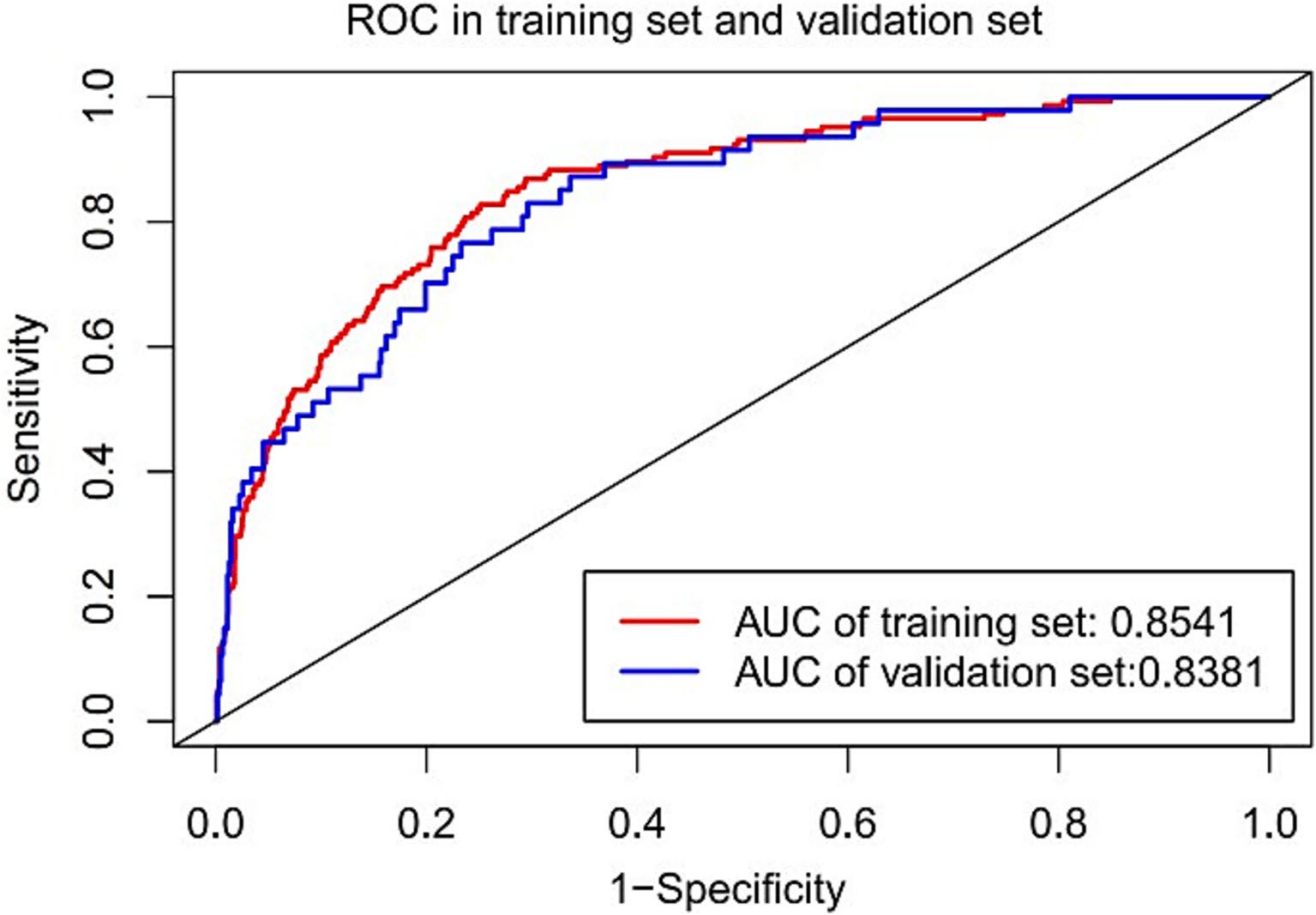
The results of ROC curve analysis in the training set and the validation set.

**Figure 5:**
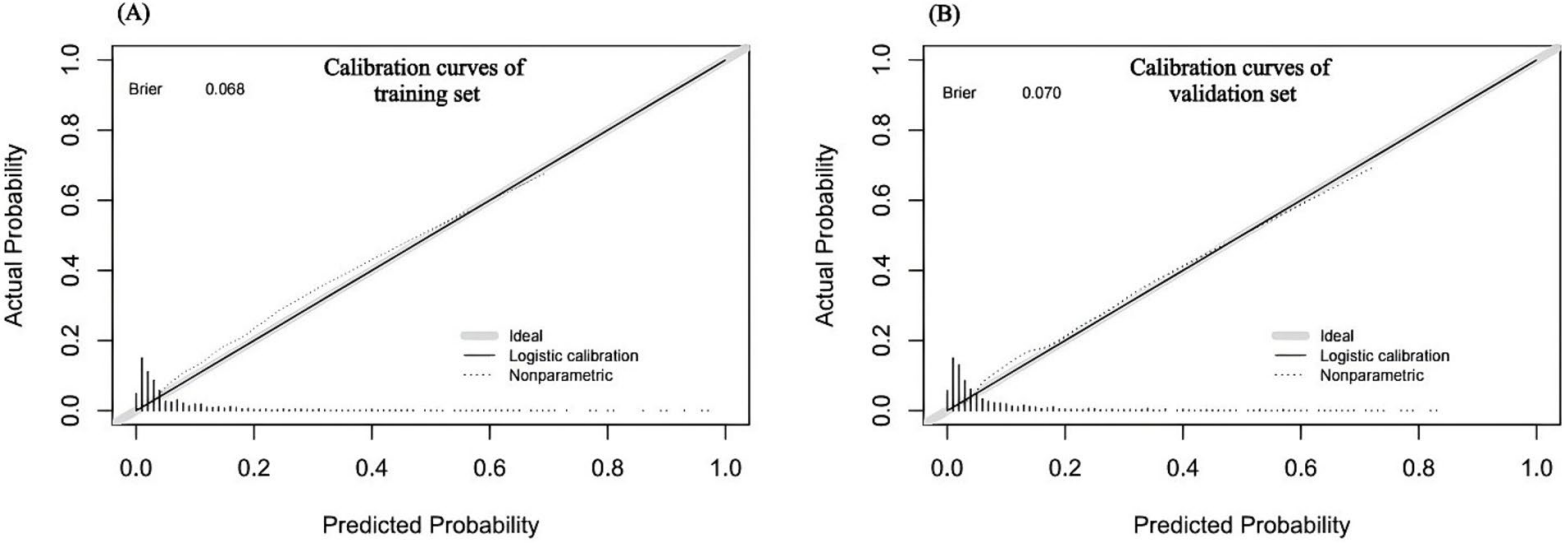
**(A)** The results of the calibration curve analysis in the training set; **(B)** The results of the calibration curve analysis in the validation set.

**Figure 6:**
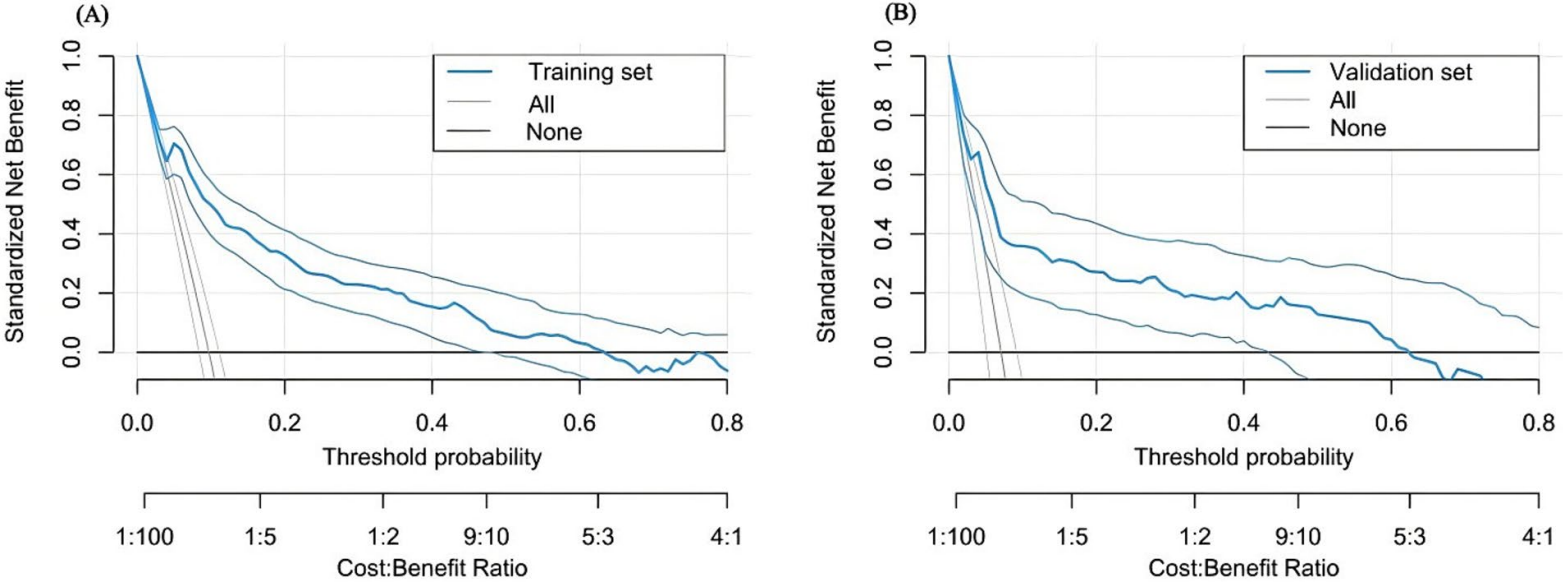
**(A)** The results of the decision curve analysis in the training set; **(B)** The results of the decision curve analysis in the validation set.

The nomogram’s discrimination ability was assessed using the receiver operating characteristic (ROC) curve. The area under the curve (AUC) was 0.8541 (95% CI: 0.833 to 0.911) in the training set and 0.8381 (95% CI: 0.752 to 0.863) in the validation set (Figure 4). Calibration curves were generated through 1,000 repetitions of bootstrap resampling (Figure 5), demonstrating excellent consistency between predicted probabilities from calibration curves and actual probabilities within both groups. The Brier score was 0.068 in the training set and 0.070 in the validation set. Decision curve analysis (DCA) was performed using R software to assess the clinical utility of this nomogram in both the training and validation sets (Figure 6). The advantage of DCA lies in its ability to enhance prediction accuracy compared to a baseline strategy, which involves not using any model. The calculation of high-risk probability in the DCA typically involves selecting appropriate thresholds for the model’s predicted outcomes. The blue decision curve shows the change in net benefit across different probability threshold values. The bottom black horizontal line labeled “None” represents the zero net benefit of not treating at all, while the gray curve labeled “All” represents the change in net benefit when treating all patients. Within a certain range, the higher the net yield of the model, the greater its clinical utility. Based on the analysis of DCA curves, it was concluded that this nomogram exhibits favorable net gain.

## Discussion

We developed a nomogram for predicting the risk of gastrointestinal bleeding (GIB) in traumatic brain injury (TBI) patients based on their demographic information, laboratory test indicators, and complications. Feature selection identified six risk factors—gender, SOFA score, shock index (SI), blood urea nitrogen (BUN), albumin level, and diabetes status—as important predictors of GIB in ICU-admitted TBI patients. The area under the curve (AUC) of the nomogram model, based on these variables, exceeded 0.8 in both the training and validation cohorts, indicating favorable prediction accuracy. This study represents the first effort to develop a user-friendly nomogram as well as a convenient predictive tool for assessing GIB occurrence in TBI patients during their ICU stay. To our knowledge, this is the first TBI-specific prediction model that integrates high-dimensional ICU data from MIMIC-IV with machine learning algorithms to generate an actionable nomogram for GIB prevention. We anticipate that this model will facilitate the development of more personalized treatment and intervention strategies.

In this study, 50 out of 2,774 TBI patients developed acute GIB, accounting for 1.8%. A previous study (18) reported that the incidence of acute gastrointestinal injury in ICU patients was 51.7%, indicating that critically ill patients are at higher risk of developing such injuries. Severe craniocerebral trauma can disrupt the central nervous system’s regulation of gastrointestinal function, particularly in the hypothalamus and brainstem regions. This disruption leads to an imbalance in the autonomic nervous system, which subsequently affects gastrointestinal motility, secretion, and absorption, potentially resulting in gastrointestinal dysfunction (19, 20). Moreover, prolonged immobility and mechanical ventilation may reduce gastrointestinal motility, leading to gastric retention and gastroesophageal reflux, which further exacerbate gastric mucosal damage and increase the risk of acute gastrointestinal injury (21, 22). Additionally, severe trauma, sepsis, and shock can trigger stress responses that compromise intestinal endothelial integrity, leading to increased intestinal permeability and contributing to acute gastrointestinal injury (23).

So far, some studies have investigated the role of albumin in young and middle-aged patients with cerebral hemorrhage who experienced postoperative GIB complications (24). The incidence of GIB within 14 days after admission was significantly lower in the high albumin group compared to the low albumin group [12.5% (4/32) vs. 31.3% (10/32), *p* < 0.05]. These findings suggest that improving nutritional status can significantly reduce the occurrence of postoperative gastrointestinal complications, promote neurological recovery, and improve long-term prognosis. Consistent with these results, our study demonstrated that a low albumin level is strongly associated with higher rates of GIB following traumatic brain injury (TBI). Several studies have reported on prognostic factors in patients with GIB, including serum BUN, creatinine, and albumin levels (25, 26). While an increase in serum BUN may result from dehydration, azotemia can also occur in GIB patients due to the absorption of blood products in the gastrointestinal tract (27). Bae et al. (28) reported that the blood urea nitrogen to serum albumin ratio (B/A ratio) is an independent predictor of poor prognosis in geriatric patients with GIB.

The possible mechanisms by which diabetes mellitus contributes to gastrointestinal bleeding (GIB) can be summarized in the following three aspects: (1) In individuals with prediabetes, microvascular and macrovascular lesions are already present (29). Oxidative stress, enhanced polyol metabolism, and activation of protein kinase C further exacerbate microvascular damage, leading to gastric mucosal injury and subsequent bleeding (30); (2) In patients with diabetes, the increased release of pro-inflammatory cytokines inhibits angiogenesis, resulting in delayed tissue healing and an elevated risk of GIB (31); (3) Both prediabetic and diabetic patients often experience neuropathy, which may dull pain perception and delay diagnosis and treatment, allowing gastric mucosal injury to progress more readily to GIB (32). In our study, patients with diabetes were found to have a higher susceptibility to developing GIB, with an odds ratio (OR) of 1.279894.

Our research findings indicated that the shock index (SI) was an independent risk factor for gastrointestinal bleeding (GIB), with an odds ratio (OR) of 1.16. When the shock index exceeds 1.0, microcirculation in vital organs and tissues becomes insufficient, leading to metabolic disorders that fail to meet the body’s normal physiological needs (33). A decrease in total blood volume by 10% does not initially affect heart rate, blood pressure, or cardiac output but can reduce hepatic and gastrointestinal blood flow by up to 40% (34). This redistribution of blood flow throughout the body causes a relative reduction in intestinal blood flow, resulting in ischemia and hypoxia of the intestinal mucosa, destruction of microcirculation, and oxidative stress injury, which further damages the intestinal mucosa. Gastrointestinal mucosal ischemia and hypoxia are the primary pathological basis for gastrointestinal dysfunction. It has been reported that severe trauma or stress suppresses the function of the mononuclear-macrophage system (35), potentially increasing the absorption of endotoxins by the gastrointestinal mucosa. Furthermore, ischemia and necrosis of the gastrointestinal tract damage the barrier function, releasing large amounts of endotoxins into the bloodstream. These endotoxins trigger the release of toxic mediators such as oxygen free radicals, TNF-*α*, platelet-activating factor (PAF), IL-1, and IL-6 (36), which further impair gastrointestinal mucosal circulation, exacerbate intestinal mucosal barrier damage, and ultimately lead to GIB.

This study established and validated a predictive model for gastrointestinal bleeding (GIB) risk in intensive care unit (ICU) patients with traumatic brain injury (TBI), which has significant clinical implications. Firstly, the research identified independent risk factors associated with GIB, including gender, Sequential Organ Failure Assessment (SOFA) score, shock index (SI), blood urea nitrogen (BUN), albumin levels, and diabetes. These findings provide valuable therapeutic insights for clinicians. By recognizing these pertinent risk factors, healthcare providers can implement preventive measures and treatment strategies aimed at reducing the incidence of GIB and improving patient outcomes. Secondly, the predictive nomogram serves as a valuable tool for risk stratification and informed decision-making in clinical practice. Healthcare providers can use the nomogram to calculate individualized risk scores for TBI patients, facilitating early identification and targeted intervention for those at high risk.

We acknowledge that this study has certain limitations. First, while SOFA (*p* = 0.029), SI (*p* = 0.050), and diabetes (*p* = 0.05) demonstrated marginal significance in univariate analysis, their inclusion in the final model was justified by their non-zero coefficients in the LASSO regression, which indicates their independent predictive value despite their borderline *p*-values. Second, as a retrospective cohort study, our findings may be subject to bias due to the exclusion of variables related to vital signs and biochemical indicators when the percentage of missing values exceeded 15% of the total sample size. Third, although internal validation was performed using this database, external validation is required to confirm the robustness and generalizability of the nomogram. Lastly, owing to the limited range of variables available in the public database, several clinically significant factors, such as subgroup analyses of brain injury types and lactate levels, were not included in the analysis.

## Conclusion

In summary, the predictive nomogram developed in this study addresses a significant gap in clinical practice within the field, offering clinicians an effective tool for assessing the risk of gastrointestinal bleeding (GIB) in patients with traumatic brain injury (TBI) admitted to the intensive care unit (ICU). The early identification of high-risk patients and subsequent targeted interventions can enhance patient outcomes and mitigate the burden of GIB among vulnerable populations. Nevertheless, further research and validation are essential to confirm the utility and applicability of this nomogram.

## Data Availability

The raw data supporting the conclusions of this article will be made available by the authors, without undue reservation.
